# Long Life

**Published:** 1856-11

**Authors:** 


### LONG LIFE.

The physiological law of animal existence is, that the duration of life should be at least five fold that of growth. The horse is four or five years attaining his full growth, and lives twenty-five years. The ox lives fifteen, and the dog ten years. The cat lives six times the growing period; the rabbit eight. Men usually attain their growth at about twenty years of age, and yet, comparatively few, reach four score years. More than one-half of all. who are born, do not attain the age of twenty.

Being made to live an hundred years, it is a sad reflection, that nine-tenths die before they reach the half-way house; before half the work of life is done. This result is owing to three main causes:—

First. To artificial modes of life.

Second. To over-indulgence of the appetites and passions of our nature.

Third. To the wearing ambition, to the wasting anxieties, to the depressing cares of life.

A cultivated intelligence, and a well informed conscience, and these only, are competent to remove these causes of the premature decay of our race. But mark—a man must be conscientous, as well as intelligent. He must be wise to know what is duty; he must be moral, to impel him to its discharge.

The secret of long life is given in the short history of one who, in his eighty-fourth year, was the picture of a mellow old age, and bade fair to live twenty years longer. SHARON CARTER, of Philadelphia, at that great age, had rarely been sick. His life was one of industrious out-door activities. He traveled much, always on foot, slept with his window wide open, in all kinds of weather, and maintained a cheerful equanimity.

Therefore, in the beautiful language of that ripe medical scholar, Dr. Thompson, of London,—Let our education be so conducted, as to train the mind for tranquil superiority to passing cares, and to qualify for the exhilarating occupations of a useful life.

